# The Population Impact of a Large School-Based Influenza Vaccination Campaign

**DOI:** 10.1371/journal.pone.0015097

**Published:** 2010-11-30

**Authors:** Carlos G. Grijalva, Yuwei Zhu, Lone Simonsen, Edward Mitchel, Marie R. Griffin

**Affiliations:** 1 Department of Preventive Medicine, Vanderbilt University School of Medicine, Nashville, Tennessee, United States of America; 2 Department of Biostatistics, Vanderbilt University School of Medicine, Nashville, Tennessee, United States of America; 3 Department of Global Health, George Washington University, Washington, D.C., United States of America; 4 Department of Medicine, Vanderbilt University School of Medicine, Nashville, Tennessee, United States of America; University of Hong Kong, Hong Kong

## Abstract

**Background:**

The optimal vaccination strategy to mitigate the impact of influenza epidemics is unclear. In 2005, a countywide school-based influenza vaccination campaign was launched in Knox County, Tennessee (population 385,899). Approximately 41% and 48% of eligible county children aged 5–17 years were immunized with live attenuated influenza vaccine before the 2005–2006 and 2006–2007 influenza seasons, respectively. We sought to determine the population impact of this campaign.

**Methods:**

Laboratory-confirmed influenza data defined influenza seasons. We calculated the incidence of medically attended acute respiratory illness attributable to influenza in Knox and Knox-surrounding counties (concurrent controls) during consecutive seasons (5 precampaign and 2 campaign seasons) using negative binomial regression and rate difference methods. Age-stratified analyses compared the incidence of emergency department (ED) visits and hospitalizations attributable to influenza.

**Results:**

During precampaign seasons, estimated ED visit rates attributable to influenza were 12.39 (95% CI: 10.34–14.44) per 1000 Knox children aged 5–17 years and similar in Knox-surrounding counties. During the campaign seasons, annual Knox influenza-associated ED visit rates declined relative to rates in Knox-surrounding counties: rate ratios 0.55 (95% CI: 0.27–0.83) and 0.70 (95% CI: 0.56–0.84) for the first and second campaign seasons, respectively. Overall, there were about 35% or 4.86 per 1000 fewer influenza-associated ED visits among Knox County children aged 5–17 years attributable to the campaign. No significant declines in Knox compared to surrounding counties were detected for influenza associated ED visits in children aged <5 years, all adults combined or selected adult age subgroups, although power for these analyses was limited. Alternate rate-difference analyses yielded consistent results.

**Conclusion:**

Vaccination of approximately 45% of Knox school-aged children with influenza vaccine was associated with a 35% annual reduction (4.86 per 1000) in ED visit rates attributable to influenza. Higher vaccination coverage and/or larger studies would be needed to determine whether similar interventions have indirect benefits in other age groups.

## Introduction

The optimal strategy to mitigate the population health impact of influenza epidemics is unclear. Safe and effective vaccines are the cornerstone for prevention of both seasonal and pandemic influenza; however, coverage remains suboptimal in the US despite long-standing recommendations for targeted vaccination of high risk groups and seniors, and the recent expansion to universal vaccination.[1], [2], [3], [4]

Children are crucial transmitters of influenza virus within communities.[5], [6] School-aged children have high influenza attack rates, are infected early in the course of epidemics, shed viruses for longer periods than adults and have a higher intensity of social contact than other age groups.[5], [7], [8], [9] Therefore, targeted vaccination of school-aged children, especially when there is a good match between vaccine and circulating strains, could reduce the impact of epidemics by preventing both influenza disease in vaccinated children and also transmission of the virus to other age groups.

Live attenuated influenza vaccines (LAIVs) are attractive for school-based interventions because compared to inactivated vaccines, they have higher efficacy in young children,[10], [11], [12] result in reduced virus shedding among vaccinees after influenza infection,[13] and their “needle-less” mode of administration has high acceptability.[11], [14], [15] School-based vaccination campaigns with LAIV have been reported to reduce influenza illness among vaccinees,[16], [17], [18] reduce influenza-like illnesses among their close contacts, and modestly reduce school absenteeism.[16], [17] Although mathematical models and experimental evidence suggest benefits of such targeted vaccination strategies among unvaccinated persons in selected populations (i.e. indirect or herd protection), [9], [19], [20], [21], [22], [23] consistent evidence of herd protection is limited.[14], [16], [24]

In 2005, Tennessee's Knox County Health Department launched a school-based influenza vaccination campaign with the aim of immunizing public school children from kindergarten through 12th grade. From October-December 2005, LAIV was offered through weekday in-school and weekend clinics. Children with signed parental consent forms and without known contraindications to LAIV were vaccinated. MedImmune donated LAIV and other campaign costs were assumed by the Knox County Health Department.[18], [25], [26], [27] Prior to the 2005–2006 influenza season, 24,198 (45%) of 53,420 Knox public school students were immunized with LAIV. Approximately 58% of 5,099 children aged 5–9 years received a second dose. In the following 2006–2007 campaign year, MedImmune donated LAIV and additional resources to strengthen vaccination efforts and to extend the campaign to private schools. Prior to the second (2006–2007) season, 47% of 54,786 public school children and 61% of 5,998 private school children were immunized. Approximately 53% of children aged 5–9 years received a second dose. The estimated overall coverage of eligible Knox school children aged ≥5 years was 41% and 48% for the 2005–2006 and 2006–2007 influenza seasons, respectively.[18], [25], [26], [27]

The second year of the Knox vaccination campaign (2006–2007) was evaluated in two studies that compared influenza activity in Knox with that of another urban Tennessee County. These studies suggested that the campaign resulted in a reduction in the proportion of influenza associated ED visits in school-aged children and influenza hospitalizations in adults aged 50 to 64 years.[25], [26] A third study reported that compared with children from surrounding counties, Knox County children had a reduced proportion of positive rapid influenza tests during the campaign seasons (2005–2007).[18] Nevertheless, none of these studies could quantify the campaign effects on actual ED and hospitalization rates among school-aged children, and the effects on non-targeted age groups remains unclear. In the present study we assessed the population impact of the two-year countywide Knox influenza vaccination campaign.

## Methods

### Ethics Statement

This study was approved by the Institutional Review Boards of Vanderbilt University and the East Tennessee Children Hospital, and by the State of Tennessee Department of Health. No informed consent was needed for this retrospective study of information already collected (See Healthcare encounters data section).

### Overview

We performed a retrospective cohort study to estimate the effectiveness of the Knox influenza vaccination campaign (2005–2007) using data from seven consecutive influenza seasons (2000–2001 through 2006–2007). Eight counties surrounding Knox (Knox-surrounding counties), which had laboratory influenza activity data and related healthcare utilization similar to Knox during the five consecutive pre-campaign seasons, served as concurrent controls.[28]

### Laboratory-confirmed influenza infections and influenza seasons

Results of rapid influenza tests from East Tennessee Children's Hospital, the regional hospital serving children from Knox and Knox-surrounding counties, were used to define seven consecutive influenza seasons, 2000–2001 through 2006–2007. Each influenza season began the week when the cumulative proportion of positive rapid influenza tests observed during that winter (November through April) reached 2.5%, and ended the week the cumulative proportion reached 97.5%. Thus, each season contained at least 95% of all positive influenza tests from that season.[18] Using the influenza seasonal information, we also defined all non-influenza winter weeks as peri-Influenza season.[29]

### Healthcare encounters data

We identified healthcare encounters using the electronic Tennessee Hospital Discharge Data System, which includes data on hospitalizations and emergency department (ED) visits from all Tennessee Department of Health-licensed hospitals. This information is collected systematically for administrative purposes by Tennessee Law mandate. Up to 9 diagnoses per record are coded using International Classification of Diseases - Clinical Modification-Ninth Revision (ICD9-CM) codes.

### Study outcomes

We assessed the effects of the vaccination campaign on the excess of medically attended acute respiratory illness (MAARI) observed during influenza seasons, that is, the incidence of MAARI attributable to influenza above seasonal baseline. MAARIs included diagnoses (in any position) of otitis media, sinusitis, lower and upper respiratory tract disease and fever (ICD9-CM: 381–383, 460–466, 480–487,490, 491, 493 and 7806).[14], [30], [31], [32] All MAARI hospitalizations and ED visits were mutually exclusive, and subjects could contribute a maximum of one healthcare encounter per setting per week, such that an ED visit and a hospitalization on the same day qualified only as a hospitalization.

### Statistical Analyses

Weekly MAARI rates were calculated using the weekly number of MAARI, and population estimates obtained from the US Census Bureau.[33] School-aged children (5–17 years old) were the target population of the vaccination campaign and thus, the primary population for this evaluation. Indirect (herd) protection was assessed in other age groups: <5, 18–49, 50–64 and 65 or more years. Furthermore, since vaccination uptake was slightly higher among children attending kindergarten/elementary schools than among those attending middle/high school[18], separate estimates were obtained for children aged 5–11 and 12–17 years. We also obtained separate estimates for adults aged 18–34 and 35–49 years old, as social contact patterns suggest that this second group has higher intensity of contacts with school-aged children [8].

To estimate weekly MAARI attributable to influenza (excess MAARI) we first estimated the weekly rates that would be expected in the absence of influenza. This baseline was calculated using age-specific count-event models that included indicators for calendar week, cyclical terms to account for seasonality and one indicator for influenza activity (proportion of positive rapid influenza tests)[34], [35], [36]. Since Poisson models showed data overdispersion, we used more robust negative binomial models for all estimations and allowed for non-linearity of the weekly time term using restricted cubic splines. Using these models' coefficients, we set the influenza indicator to 0 for all observations and estimated the predicted baseline weekly MAARI rates, that is, the rates that would be expected in the absence of influenza activity.[37], [38] Excess MAARI was calculated as the difference between observed MAARI weekly rates and the baseline predicted rates during influenza seasons. Excess MAARI was set to 0 for those weeks where predicted baseline exceeded the observed incidence.[29], [34], [39]

In the secondary rate difference analysis,[29], [34] age-specific excess MAARI was estimated subtracting baseline estimates from the observed values. We used MAARI rates during peri-influenza seasons as the baseline over which we estimated the excess MAARI.[34] Rate differences were set to 0 for those seasons where the peri-Influenza season estimates exceeded the influenza season estimates.[29], [34], [39]

Seasonal excess MAARI rates and 95% confidence intervals were estimated separately for Knox and Knox-surrounding counties, and compared using rate ratios.[18] Ninety-five percent confidence intervals for these excess rate ratios were calculated using the Delta method,[40] and negative estimates were truncated at 0. Finally, the Knox campaign effectiveness was estimated as (1-rate ratio) ×100% for each of the two campaign seasons. All analyses were stratified by setting (ED visits and hospitalizations). Statistical analyses were performed using SAS 9.1.3 and Stata 11.

## Results

### Study populations

The US Census Bureau estimated Knox County and Knox-surrounding counties (eight counties) populations to number 385,899 and 422,064 people, respectively in 2000. Both populations were predominantly white (88.7% vs. 96.1%), children aged 5–17 years accounted for 16.2% and 17% of the two populations, and 82.5% and 71.2% of those aged ≥25 years attained high school graduation or higher education (Table 1).

**Table 1:** Selected demographic characteristics of Knox and Knox-surrounding counties.

	Knox County	Knox-surrounding counties*
Total population (n)	385,899	422,064
**Age groups (%)**		
<5	6.1	5.9
5 to 17	16.2	17.0
18 to 49	49.5	44.3
50 to 64	15.6	18.4
65 or more	12.6	14.4
**Gender (%)**		
Female	51.7	51.4
**Race (%)**		
White	88.7	96.1
Black	8.7	2.2
Other	2.6	1.7
**Enrolled in school (aged ≥3 years) (%)**		
Nursery school, preschool	6.1	5.1
Kindergarten	4.7	5.7
Elementary school (grades 1–8)	37.8	50.5
High school (grades 9–12)	18.3	23.5
College or graduate school	33.1	15.3
**Educational attainment (aged ≥25 years) (%)**		
High school graduate or higher	82.5	71.2
Bachelor's degree or higher	29	13.8
**Income**		
Median household income (US$)	37,454	33,731
Families below poverty level (%)	8.4	10.6

*Data are from the US Census Bureau for year 2000.

### MAARI ED visits and hospitalizations

During the seven consecutive winter seasons, there were 30630 MAARI ED visits and 1395 MAARI hospitalizations among Knox children aged 5–17 years. In Knox-surrounding counties, the numbers of ED visits and hospitalizations were 36670 and 1678, respectively. The average number of weeks in each laboratory defined-influenza season was 13 (range: 10–16 weeks).

### Influenza activity

**Figure 1:**
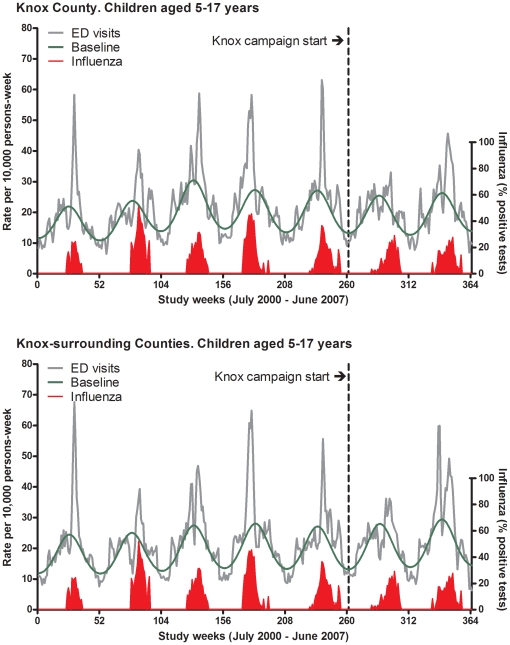
Influenza activity among school-aged children, Emergency Department (ED) visits, 2000–2007. Footnote: The green line represents the model predicted baseline rates. That is, the rates that would be expected in the absence of influenza activity.

In both Knox and Knox-surrounding counties, MAARI ED visit rates among children aged 5–17 years consistently peaked during the influenza seasons (Figure 1).

For children aged 5–17 years, MAARI ED visit rates attributable to influenza (excess MAARI ED visits) in Knox County ranged from 4.6 per 1000 to 17.1 per 1000, and from 7.1 per 1000 to 19.4 per 1000 in Knox surrounding counties. MAARI hospitalization rates attributable to influenza in Knox County ranged from 0.2 to 0.5 per 1000, and from 0.3 to 0.6 per 1000 in Knox-surrounding counties.

MAARI ED visits and hospitalization rates attributable to influenza were similar in Knox and Knox-surrounding counties during the pre-campaign seasons (Tables 2 and 3).[28]

**Table 2:** Ratios of excess MAARI rates of ED visits attributable to influenza (Knox/Knox-surrounding counties).

	Pre-Campaign seasons			Campaign seasons		
Age group	Knox rate/1000	Knox-surrounding rate/1000	Excess Rate ratio (95% CI)	Knox rate/1000	Knox-surrounding rate/1000	Excess Rate ratio (95% CI)
<5 years	38.73	40.18	0.96 (0.73, 1.19)	30.18	33.72	0.90 (0.60, 1.20)
5 to 17 years	12.39	13.02	0.95 (0.74, 1.16)	9.11	13.97	**0.65 (0.46, 0.84)** *
5 to 11 years	14.78	14.87	0.99 (0.71, 1.27)	11.71	17.50	**0.67 (0.44, 0.90)** *
12 to 17 years	9.97	11.16	0.89 (0.59, 1.19)	6.72	10.12	**0.66 (0.34, 0.98)** *
18 to 49 years	4.44	5.22	0.85 (0.63, 1.07)	2.95	3.52	0.84 (0.48, 1.20)
18 to 34 years	5.55	7.33	**0.76 (0.53, 0.99)** *	3.64	4.69	0.78 (0.38, 1.18)
35 to 49 years	3.32	3.48	0.95 (0.51, 1.39)	2.48	2.61	0.95 (0.31, 1.59)
50 to 64 years	2.92	3.07	0.95 (0.46, 1.44)	2.35	2.04	1.15 (0.23, 2.07)
65 or more years	3.38	3.46	0.98 (0.49, 1.47)	2.42	2.44	0.99 (0.21, 1.77)
18 or more years	3.90	4.31	0.90 (0.71, 1.09)	2.66	2.83	0.94 (0.61, 1.27)

Footnote: *Indicate significant change. Excess rate ratio 95% confidence intervals (CI) did not include 1.

**Table 3:** Ratios of excess MAARI rates of hospitalization attributable to influenza (Knox/Knox-surrounding counties).

	Pre-Campaign seasons			Campaign seasons		
Age group	Knox rate/1000	Knox-surrounding rate/1000	Excess Rate ratio (95% CI)	Knox rate/1000	Knox-surrounding rate/1000	Excess Rate ratio (95% CI)
<5 years	3.15	3.98	0.79 (0.12, 1.46)	2.17	2.58	0.84 (0, 1.94)
5 to 17 years	0.37	0.45	0.82 (0, 1.98)	0.41	0.44	0.93 (0, 2.38)
5 to 11 years	0.45	0.57	0.79 (0, 2.13)	0.47	0.56	0.84 (0, 2.31)
12 to 17 years	0.44	0.43	1.02 (0, 2.69)	0.53	0.57	0.93 (0, 2.56)
18 to 49 years	0.39	0.49	0.80 (0, 1.63)	0.27	0.49	0.55 (0, 1.46)
18 to 34 years	0.46	0.41	1.12 (0, 2.53)	0.43	0.54	0.8 (0, 1.91)
35 to 49 years	0.39	0.68	0.57 (0, 1.43)	0.30	0.58	0.52 (0, 1.76)
50 to 64 years	1.3	1.75	0.74 (0.04, 1.44)	1.14	1.38	0.83 (0, 1.96)
65 or more years	3.75	4.49	0.84 (0.33, 1.35)	2.95	3.32	0.89 (0.05, 1.73)
18 or more years	1.02	1.49	0.68 (0.34, 1.02)	0.63	1.13	0.56 (0.06, 1.06)

### Effectiveness of the Knox Vaccination campaign

**Figure 2:**
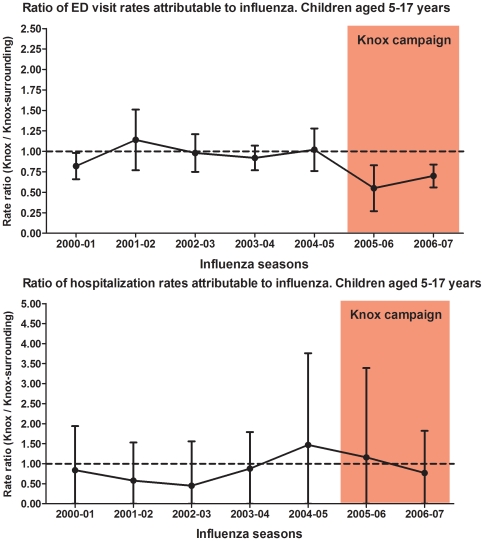
Estimated rates and rate ratio of Emergency Department (ED) visits and hospitalization rates attributable to influenza among school-aged children from Knox and Knox-surrounding counties.

During the campaign years, the ratio of ED visit rates attributable to influenza among children aged 5–17 years (target population) declined significantly, indicating that ED rates attributable to influenza in Knox decreased compared with the rates in Knox-surrounding counties. The ratio of rates in Knox and Knox-surrounding counties was 0.55 (95% CI: 0.27–0.83) and 0.70 (95% CI: 0.56–0.84) during the first and second year of the vaccination campaign, respectively (Figure 2).

Overall, the ratio of average ED visit rates during the pre-campaign seasons (2000–2005) and during the campaign seasons (2005–2007) was 0.95 (0.74, 1.16) and 0.65 (0.46, 0.84), respectively (Table 2). During the campaign, the average influenza associated ED rates in Knox were 9.11 (95% CI: 6.94, 11.28) compared to 13.97 (95% CI: 11.79, 16.15) per 1000 in Knox-surrounding counties. Similar results were observed among children aged 5–11 and 12–17 years old (Table 2). Hence, the estimated overall effectiveness of the vaccination campaign in preventing influenza-associated ED visits among children aged 5–17 years was estimated to be 35% (95% CI: 16–54), translating to 4.86 fewer ED visits per 1000 Knox County children annually.

MAARI hospitalization rates attributable to influenza among children aged 5–17 years from Knox and Knox-surrounding counties were similar during the pre-campaign and campaign seasons. The rate ratios remained relatively stable and were not different from 1 throughout the study period. The ratios of average hospitalization rates during the pre-campaign and campaign seasons were not significantly different from 1. Similar results were observed among children aged 5–11 and 12–17 years old (Table 3).

MAARI incidence attributable to influenza among other age groups not targeted by the Knox campaign (children aged <5 years, all adults combined and selected adult age subgroups) remained similar throughout the study years in both Knox and in Knox-surrounding counties. In these groups, rate ratios of ED visit rates and hospitalizations attributable to influenza for Knox and Knox-surrounding counties were not different from 1 (Tables 2 and 3).

Results from the rate difference analysis were consistent with the primary analysis, indicating that rates of ED visits attributable to influenza in the target population (children aged 5–17 years) were 0 and 6.1 per 1000 (rate ratio could not be computed) during the first campaign season for Knox and Knox-surrounding counties, respectively. During the second campaign season rates were 14.8 and 20.7 per 1000 (rate ratio 0.71, 95% CI: 0.58–0.84). Results for non-targeted age groups were, in general, similar to those from the primary analyses (See Supporting Information Tables S1 and S2).

## Discussion

Our findings indicate that the large Knox school-based influenza vaccination campaign was associated with a significant reduction in morbidity attributable to influenza. These declines coincided temporally with the vaccination campaign and were observed exclusively in the 5–17 year old target population of the campaign, with consistent results observed in secondary analyses. Nevertheless, our study had limited power to detect significant declines among non-targeted age groups, and none were detected.

The incidence of MAARI ED visits attributable to influenza declined in the target population, indicating a direct protection of the vaccination campaign. No significant effects were observed on MAARI hospitalizations but these events were rare, and despite the large sample size of our study, we could not rule out sizable campaign effects on this outcome. Based on the analyses of data from pre-campaign years for the target population, we previously estimated that our study would be powered to detect a 20% decline in ED visits rates, but a much larger 91% decline in hospitalization rates.[28]

Although previous studies suggested that indirect protection of unvaccinated groups against influenza-like-illness and reductions in absenteeism could be achieved through vaccination of school-aged children,[14], [15], [16], [17], [41], [42] we were unable to show significant disease reductions among age groups that were not directly targeted during the Knox vaccination campaign. It is possible that the influenza vaccination coverage among school children (approximately 45%) was not high enough to achieve significant protective levels in other groups. A previous study reported significant indirect protection against MAARI among adults aged ≥35 years with even lower vaccination coverage of children aged 1.5 through 18 years (e.g. approximately 25%, 35% and 50% coverage during three consecutive influenza seasons).[14] However, in that study significant protection was not demonstrated among children or younger adults who were vaccinated.[14] It is important to note that most contemporary school or community-based influenza studies have achieved vaccination coverage of 50% or lower.[14], [15], [16] The feasibility of achieving higher vaccination coverage through promotion or education campaigns deserves further scrutiny.

Another factor limiting the ability of this study to demonstrate indirect effects could be vaccination coverage among non-targeted age groups. Prior studies showed parent/self-reported vaccination rates in Knox County residents of 36% for children aged <5 years and 70% for adults aged 50 years or older.[25], [26] Detecting indirect protection in populations where influenza vaccination is now universally recommended will be challenging, and limited to reductions in the residual disease burden in those populations. A recent article assessed the effect of school-based influenza vaccination on healthcare encounters in the Province of Ontario, Canada (2000–2007). This province has had a universal influenza vaccination program since year 2000. Within this context, Kwong et al[43] compared the incidence of influenza associated healthcare encounters between public health units (PHUs) that had school-based influenza vaccination available in at least 50% of elementary and secondary schools, with PHUs without such availability. Vaccination coverage in children in secondary schools was 39% in PHUs with and 30% in those without vaccination programs available. There was a modest reduction in the frequency of physician office visits for influenza-associated pneumonia and influenza in children attending secondary schools in PHUs with vaccination programs compared to those without such programs (estimated vaccine effectiveness: 19%), but no significant reductions in emergency department visits or hospitalizations among school aged-children. There were no significant differences in visits for the more common influenza-associated acute respiratory illnesses in the targeted age group; and, similar to our study, no benefits were detected in other non-targeted groups.[43]

A recent cluster randomized clinical trial demonstrated that vaccination of 83% of children aged 3–15 years in Hutterite colonies significantly reduced the incidence of influenza illness in both immunized children and their unvaccinated close contacts. Although the study also reported non-significant declines in physician visits for respiratory diseases and influenza like-illnesses in colonies randomized to influenza vaccines, the trial was not adequately powered to assess these outcomes.[20] The very high vaccine coverage achieved, the particular demographics and low background vaccination coverage in the study communities likely contributed to the observed indirect protection. Additional studies in populations with low background vaccination rates may help quantify the minimum vaccination coverage required to attain measureable indirect benefits.[24]

The identification of a proper comparison population had implications for our assessment of effectiveness. Previous studies have demonstrated substantial differences in the seasonal incidence of influenza-related healthcare encounters between different communities.[44] Our assessment of the campaign effectiveness used Knox-surrounding counties as comparison because in spite of minor demographic differences between study populations (Table 1), the incidence of healthcare utilization attributable to influenza was similar in five consecutive pre-campaign influenza seasons.[28]

The two different analytical approaches used to estimate MAARI attributable to influenza found similar estimates.[34] Our analyses suggest that the overall effectiveness of the Knox campaign in preventing ED visits among children aged 5–17 years was 35% (95% CI: 16–54). This estimate encompassed both direct and indirect protection in this age group,[43] and suggests that the Knox vaccination campaign prevented approximately 300 influenza ED visits in this target population during each influenza season. Using the estimated effectiveness, we also calculated crude estimates of vaccine efficacy for Knox children aged 5–17 years. Taking into account that ∼45% of all Knox school-aged children were immunized, the observed excess rate ratio of 0.65 and assuming that influenza rates were the same for unvaccinated children from Knox and Knox-surrounding counties, the estimated vaccine efficacy provided by LAIV against ED visits would be 77.8% (RR: 0.65 = [1−(0.45*Vaccine Efficacy)]/1). These estimates assumed no vaccination of school-aged children in Knox-surrounding counties; accounting for influenza vaccination in control communities would increase the vaccine efficacy estimates.

We used all data available for ED visits and hospitalizations for the study populations. This information was systematically collected for administrative purposes and thus, unlikely to be affected by recall issues. Study data included all hospital-based care without regard to payment sources and thus it was virtually free of selection issues. Moreover, the use of laboratory-confirmed influenza information to define influenza seasons reduced misclassification of periods with influenza activity.

Despite those strengths, the interpretation of our findings requires the consideration of several caveats. First, our data lacked virological confirmation for all observed MAARIs and potential misclassification of MAARI attributable to influenza cannot be ruled out. Nevertheless, our estimates were restricted to laboratory-confirmed influenza seasons and we consider that any residual misclassification would be similar in the two study populations. Second, despite its large sample size, our study did not detect significant changes in the non-targeted adult population combined, and had limited power to identify significant effects of the intervention on ED visits for adult age subgroups. Moreover, the limited number of hospitalizations observed reduced our ability to detect true effects on this outcome. Third, information on other potential causes of MAARI during winter months (e.g. respiratory syncytial virus [RSV]) was not specifically accounted for in our analyses. However, previously studies suggest that estimates including or excluding RSV data did not significantly affect influenza estimates.[29], [36], [45] Also, RSV activity would be of greater concern in younger age groups than among children aged 5-17 years. Nevertheless, lack of adjustment for RSV illness may have limited our ability to detect indirect protection in children aged <5 years. Finally, we did not measure influenza vaccination coverage in other age groups. High vaccination rates in seniors and other age groups may have made it difficult to demonstrate indirect effects in these groups.

In conclusion, our findings indicate that a large school-based influenza vaccination campaign with LAIV that attained 45% vaccination coverage, significantly reduced ED visit rates attributable to influenza among Knox County children aged 5–17 years, the main target of the campaign. During campaign years there were approximately 5 fewer influenza-associated ED visits per 1000 Knox children aged 5–17 years annually. Higher vaccination coverage and/or larger studies will be needed to determine the effect of similar interventions in other age groups.

## Supporting Information

Table S1 Ratios of excess MAARI rates of ED visits attributable to influenza (Knox/Knox-surrounding counties). Footnote: * Indicate significant change. Excess rate ratio 95% confidence intervals (CI) did not include 1. Estimates are based on the rate difference method.
(DOC)

Table S2 Ratios of excess MAARI rates of hospitalization attributable to influenza (Knox/Knox-surrounding counties). Footnote: * Indicate significant change. Excess rate ratio 95% confidence intervals (CI) did not include 1. Estimates are based on the rate difference method.
(DOC)
