# Supplementary material for: The Population Impact of a Large School-Based Influenza Vaccination Campaign
Source: PLoS One. 2010 Nov 30;5(11):e15097. doi: 10.1371/journal.pone.0015097 (PMC3013075; doi:10.1371/journal.pone.0015097)
Supplement: Table S1 — Ratios of excess MAARI rates of ED visits attributable to influenza (Knox/Knox-surrounding counties). Footnote: * Indicate significant change. Excess rate ratio 95% confidence intervals (CI) did not include 1. Estimates are based on the rate difference method. (DOC) [file pone.0015097.s001.doc]

| **Table S1.** Ratios of excess MAARI rates of ED visits attributable to influenza (Knox / Knox-surrounding counties) | | | | | | |
| --- | --- | --- | --- | --- | --- | --- |
|  | **Pre-Campaign seasons** | | | **Campaign seasons** | | |
| **Age group** | **Knox  rate / 1000** | **Knox-surrounding rate / 1000** | **Excess Rate ratio**  **(95% CI)** | **Knox rate / 1000** | **Knox-surrounding rate / 1000** | **Excess**  **Rate ratio**  **(95% CI)** |
| < 5 years | 46.13 | 51.34 | 0.9 (0.72, 1.08) | 29.69 | 31.87 | 0.93 (0.63, 1.23) |
| 5 to 17 years | 14.62 | 14.97 | 0.98 (0.79, 1.17) | 7.13 | 13.42 | **0.53 (0.36, 0.70)*** |
| 5 to 11 years | 18.03 | 18.22 | 0.99 (0.76, 1.22) | 10.27 | 16.82 | **0.61 (0.40, 0.82)*** |
| 12 to 17 years | 10.49 | 11.21 | 0.94 (0.62, 1.26) | 3.31 | 9.47 | **0.35 (0.06, 0.64)*** |
| 18 to 49 years | 4.79 | 5.96 | **0.80 (0.61, 0.99)*** | 2.92 | 3.08 | 0.95 (0.55, 1.35) |
| 18 to 34 years | 5.60 | 8.10 | **0.69 (0.49, 0.89)*** | 2.67 | 2.51 | 1.06 (0.24, 1.88) |
| 35 to 49 years | 3.90 | 3.98 | 0.98 (0.58, 1.38) | 3.21 | 3.61 | 0.89 (0.48, 1.30) |
| 50 to 64 years | 2.85 | 3.20 | 0.89 (0.41, 1.37) | 3.46 | 2.79 | 1.24 (0.60, 1.88) |
| 65 or more years | 3.35 | 3.88 | 0.86 (0.44, 1.28) | 2.98 | 2.95 | 1.01 (0.42, 1.60) |
| 18 or more years | 4.17 | 4.91 | 0.85 (0.68, 1.02) | 3.04 | 2.99 | 1.02 (0.72, 1.32) |

Footnote: * Indicate significant change. Excess rate ratio 95% confidence intervals (CI) did not include 1. Estimates are based on the rate difference method
