# Supplementary material for: The Population Impact of a Large School-Based Influenza Vaccination Campaign
Source: PLoS One. 2010 Nov 30;5(11):e15097. doi: 10.1371/journal.pone.0015097 (PMC3013075; doi:10.1371/journal.pone.0015097)
Supplement: Table S2 — Ratios of excess MAARI rates of hospitalization attributable to influenza (Knox/Knox-surrounding counties). Footnote: * Indicate significant change. Excess rate ratio 95% confidence intervals (CI) did not include 1. Estimates are based on the rate difference method. (DOC) [file pone.0015097.s002.doc]

| **Table S2.** Ratios of excess MAARI rates of hospitalization attributable to influenza (Knox / Knox-surrounding counties) | | | | | | |
| --- | --- | --- | --- | --- | --- | --- |
|  | **Pre-Campaign seasons** | | | **Campaign seasons** | | |
| **Age group** | **Knox  rate / 1000** | **Knox-surrounding rate / 1000** | **Excess**  **Rate ratio**  **(95% CI)** | **Knox rate / 1000** | **Knox-surrounding rate / 1000** | **Excess**  **Rate ratio**  **(95% CI)** |
| < 5 years | 4.68 | 6.13 | 0.76 (0.33, 1.19) | 4.90 | 3.66 | 1.34 (0.46, 2.22) |
| 5 to 17 years | 0.29 | 0.31 | 0.94 (0, 2.81) | 0 | 0 | *Not calculated* |
| 5 to 11 years | 0.39 | 0.36 | 1.08 (0, 3.65) | 0 | 0 | *Not calculated* |
| 12 to 17 years | 0.18 | 0.24 | 0.75 (0, 3.65) | 0.27 | 0 | *Not calculated* |
| 18 to 49 years | 0.33 | 0.43 | 0.77 (0, 1.74) | 0.10 | 0.62 | **0.16 (0, 0.74)*** |
| 18 to 34 years | 0.35 | 0.29 | 1.21 (0, 3.42) | 0.30 | 0.31 | 0.97 (0, 2.97) |
| 35 to 49 years | 0.31 | 0.56 | 0.55 (0, 1.64) | 0 | 0.91 | *Not calculated* |
| 50 to 64 years | 1.29 | 1.75 | 0.74 (0.01, 1.47) | 1.72 | 2.04 | 0.84 (0.14, 1.54) |
| 65 or more years | 4.15 | 5.12 | 0.81 (0.35, 1.27) | 3.61 | 5.17 | 0.70 (0.25, 1.15) |
| 18 or more years | 1.14 | 1.62 | 0.70 (0.37, 1.03) | 1.00 | 1.81 | **0.55 (0.26, 0.84)*** |

Footnote: * Indicate significant change. Excess rate ratio 95% confidence intervals (CI) did not include 1. Estimates are based on the rate difference method
